# Single-stage acellular dermal matrix reconstruction of defects in the nose and ears with exposed cartilage: a prospective case series

**DOI:** 10.1186/s12893-022-01859-7

**Published:** 2022-12-28

**Authors:** Yung-Hsu Lei, Shu-Hung Huang

**Affiliations:** 1 Department of Surgery, E-Da Hospital, Kaohsiung, Taiwan; 2 Division of Plastic Surgery, Department of Surgery, Kaohsiung Medical University Hospital, Kaohsiung, 807 Taiwan; 3 Department of Surgery, School of Medicine, College of Medicine, Kaohsiung Medical University, No. 100, Tzyou 1St Rd., Sanmin Dist., Kaohsiung City, 807 Taiwan; 4 Division of Plastic Surgery, Department of Surgery, Kaohsiung Municipal HsiaoGang Hospital, Kaohsiung, Taiwan

**Keywords:** Nasal reconstruction, Auricular reconstruction, Asian, Acellular dermal matrix, Cartilage exposed

## Abstract

**Background:**

The treatment of soft tissue defects with exposed cartilage after tumor excision is challenging. Local flap reconstruction causes occasional scarring, especially in non-Caucasian populations. Scar treatment requires secondary procedures for aesthetic modifications. Two-step reconstruction with an acellular dermal matrix addresses this issue and yields highly acceptable aesthetic resultsWe aimed to investigate the efficacy of an artificial dermal matrix cover using one-step reconstruction for defects with cartilage exposure.

**Methods:**

From July 2018 to September 2020, seven patients were enrolled and underwent a single-stage operation using acellular dermal matrices. Patients were followed up for at least 6 months and the size of the wound, days to heal, patient satisfaction, and scar scale scores were recorded.

**Results:**

Patients were followed up for an average of 25.7 months. The average time to heal was 23.4 days postoperatively. No hyperpigmentation, tumor recurrence, or retraction was noted. High acceptance and satisfaction with the outcome were observed in all patients.

**Conclusions:**

Single-stage reconstruction yielded high acceptance of aesthetic results similar to that in two-stage reconstruction. Less time and cost make this an effective and efficient treatment for soft tissue defects compared with traditional techniques.

## Introduction

Cartilage-exposed defects of the nose and ear result from the wide excision of tumors, trauma, or iatrogenic injuries. There are many traditional methods for treating cartilage-exposed defects of the ear and nose, including primary closure, secondary intention, skin grafting, or locoregional flaps [1–8]. Cartilage does not have the innate capability to self-repair and regenerate, increasing the difficulty of reconstruction [9].

Due to skin phototypes, Asians are prone to hyperpigmentation, hypertrophic scars, and keloids after reconstruction. In practice, primary closure is chosen when the defect is minor (≤ 1 cm) or the patient is old and does not care about the aesthetic result [10, 11].

Traditionally, locoregional and paramedian forehead flaps are the primary options for the reconstruction of wounds located on the distal nose [1, 12]. Many different surgical techniques are described in Western studies that attain good results, but retraction and scars associated with rhinoplasty are more frequent in Asia [13–15]. However, these techniques are expensive and time-intensive, reducing patient satisfaction.

A local flap is the first choice for the treatment of auricular skin defects with exposed cartilage, such as the posterior auricular flap that is used for ear reconstruction [9]. Single-stage reconstruction of the ear after excision of tumors also shows good outcomes [16]. However, Asian are prone to contractures and scars after reconstructions with flaps [10, 11].

Few studies have discussed the reconstruction of cartilage-exposed defects on the nose with secondary intention; a prior study has reported reconstruction of the nasal sidewall defects with satisfactory results [4]. Re-epithelialization by secondary intention is usually completed within 4–6 weeks [4]. However, reconstruction with secondary intention may induce unpredictable distortions in nasal appearance, and it is not favored for large defects [14, 17]. Only one reported case of nasal defect at the sidewall considered whether it is appropriate to apply this method to other sites of the nose [4].

Reconstruction with an acellular dermal matrix has been performed in the past. Daya et al. reported a case of auricular reconstruction that employed two-step reconstruction with Integra^®^ (Integra Lifesciences Corporation, Princeton, NJ, USA) and skin grafts under general anesthesia [2]. Applebaum et al. proposed a two-step reconstruction with Integra^®^ graft and full-thickness skin grafting under local anesthesia [17]. Both studies had a high acceptance regarding aesthetic results; however, despite attaining satisfactory results with two-step reconstruction of cartilage-exposed defects on the nose and ear using an acellular dermal matrix, these methods are costly and require repeated surgeries.

In this report, we present a prospective case series of cartilage-exposed defects reconstructed with an acellular dermal matrix (PELNAC^®^) in a single-stage surgery after tumor excision.

## Patients and methods

Seven patients from the same university hospital in Taiwan with defects on the nose or ear after surgical excision for tumor and malignancy were enrolled from July 2018 to September 2020. All patients signed a written informed consent, and the report was approved by the institutional review board of our university hospital (KMUHIRB-E(I)-20210190). We included patients with nasal or auricular wounds with exposed cartilages and lengths < 3 cm. We excluded those unable or unwilling to pay for the acellular dermal matrix and those aged < 18 years.

During the first consultation, we recorded the size of the wound, patients’ medical history, and present illnesses. One month after the surgery, we recorded the final biopsy report and an opinion questionnaire. The questionnaire (Table 1) recorded the following details on a five-point Likert scale: patient satisfaction of reconstruction outcome, interference of the wound with daily life, and if the patient required a wide excision of the other side, whether they would choose our methods.

**Table 1:** Opinion questionnaire recorded 1 month after the operation

Question 1	Overall satisfaction regarding the therapy				
	1	2	3	4	5
	Very dissatisfied	Dissatisfied	Neutral	Satisfied	Very satisfied
Question 2	The wound interferes with daily life				
	1	2	3	4	5
	Never	Rarely	Sometimes	Frequently	Always
Question 3	If you need to receive excision of the other site again, you will choose us				
	1	2	3	4	5
	Strongly disagree	Disagree	Undecided	Agree	Strongly agree

We evaluated the patient according to the Vancouver Scar Scale for scale vascularity, pigmentation, pliability, and height (Table 2) for approximately 2–3 months of follow-up at the outpatient clinic and recorded the results [18]. We recorded any secondary procedures for aesthetic modification 6 months after surgery.

**Table 2:** Vancouver Scar Scale

Pigmentation	Normal	0
	Hypopigmentation	1
	Hyperpigmentation	2
Vascularity	Normal	0
	Pink	1
	Red	2
	Purple	3
Pliability	Normal	0
	Supple	1
	Yielding	2
	Firm	3
	Banding	4
	Contracture	5
Height	Normal (flat)	0
	0–2 mm	1
	2–5 mm	2
	> 5 mm	3

### Acellular dermal matrix

IN our report, we selected PELNAC^®^ (PELNAC^®^, Gunze Corp., Osaka, Japan) as the acellular dermal matrix. PELNAC^®^ is a bilayer acellular dermal matrix that was approved in Japan in 1995. PELNAC^®^ is a bilayer membrane, with a top silicone film layer and a porcine collagen sponge layer made from pig tendon [19]. Due to the top silicone film layer, it requires a waiting period of approximately 2–3 weeks [20].

### Surgical procedure

We adopted a single-stage procedure without the Mohs surgery technique. After resection of the tumor and partially excised the perichondrium to obtain a negative margin in all cases, we applied the bilayer acellular dermal matrix to cover the defect.

We fixed the acellular dermal matrix to the wound bed with nonabsorbable sutures and compressed the wound with gauze. Four patients were admitted for surgery and further surveys for metastasis, and the others were discharged on the day or the next day of surgery.

### Post-surgery and follow-up

We regularly prescribed pain control with acetaminophen for a week after the surgery. Wound care was performed twice a week. During wound dressing, the patient would change the gauze and disinfect the wound with sterile normal saline. One week after the surgery, we removed the stitches and re-dressed them with Steri-Strips™ (3M, St. Paul, MN, USA) at the outpatient clinic. Patients were also followed up on the 21st or 28th day after the surgery.

### Case 1

A 48-year-old man presented with a pigmented nodule over the auditory canal of the right ear for 4 months. The tumor bled easily, and many crusts formed over the lesion. The patient came to our dermatological clinic for a skin biopsy, which revealed basal cell carcinoma. Consequently, he received a wide local excision for malignancy eradication. After excision, a 1 cm^2^ defect was covered with the bilayer acellular dermal matrix and cared for twice a week. The patient underwent the examination in the form of a metastasis survey and was discharged on postoperative day 8. The sutures were removed during hospitalization on postoperative day 7.

**Fig. 1:**
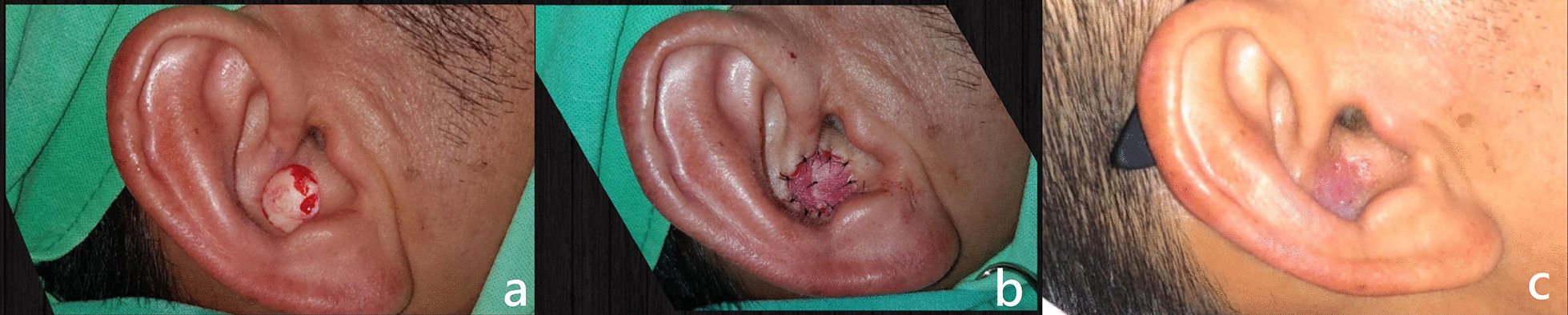
A 48-year-old man with a pigmented nodule over the auditory canal of the right ear received wide excision (**a**) and reconstruction with a bilayer acellular dermal matrix (PELNAC^®^) (**b**). The patient was followed up at the outpatient clinic on postoperative day 21 (**c**)

The patient received the first follow-up at our outpatient clinic on postoperative day 14, and the defect was healed by postoperative day 21. No further aesthetic procedures were performed, and neither contracture nor hyperpigmentation was noted. Patient satisfaction was recorded to be 4 out of 5; the Vancouver Scar Scale score was 2 (Fig. 1).

### Case 2

A 56-year-old man had a tumor on the nose for over half a year, for which he underwent a tumor biopsy. The pathology report showed basal cell carcinoma, and he subsequently underwent wide excision. A 1 cm^2^ defect was covered with the bilayer acellular dermal matrix and dressed. The patient was observed for 1 day and discharged on the next day of surgery.

**Fig. 2:**
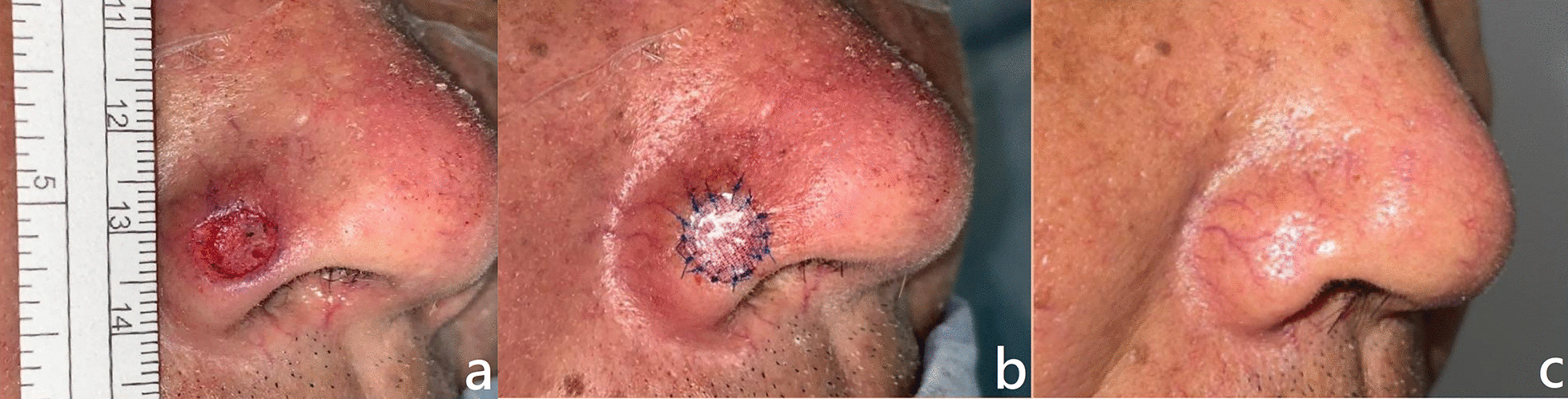
A 56-year-old man who presented with a nose tumor he noted 6 months previously received wide excision (**a**) and reconstruction with PELNAC^®^ application (**b**). The patient was followed up at the outpatient clinic 6 months postoperatively (**c**)

We removed the sutures at the first follow-up at our outpatient clinic on postoperative day 7, and the defect was healed by postoperative day 14. No further aesthetic procedures were performed, and neither contracture nor hyperpigmentation was noted. Patient satisfaction was recorded to be 4 out of 5; the Vancouver Scar Scale score was 1 (Fig. 2).

## Results

Seven patients underwent surgery (three men); the mean (range) age was 52.28 (18–75) years. Two patients had lesions on the nasal tip, two on the nasal ala, and three on the ear concha. Six patients had a pathological diagnosis of malignancy from a previous biopsy; the remaining patient’s biopsy result indicated nevus (Tables 3 and 4).

**Table 3:** Characteristics of the patients

	Age	Sex	ADM	Suture	History	Location	Biopsy	Size, cm	Healed day	Complication	Follow up period, months	Q1	Q2	Q3	Scar scale	Secondary modification
1	56	M	PELNAC®	Prolene 6–0	Hepatitis B	Right nasal ala	BCC	1 × 1	14	Nil	16	5	1	5	2	NO
2	64	F	PELNAC®	Prolene 6–0	Nil	Right ear concha	BCC	1 × 1	28	Nil	17	5	1	5	1	NO
3	67	F	PELNAC®	Prolene 7–0	HTN; Hyperlipidemia; Breast cancer; Keloid	Nasal tip	BCC	1 × 1	18	Nil	17	5	1	5	1	NO
4	38	F	PELNAC®	Nylon 6–0	Nil	Left ear concha	Compound nevus	0.5 × 0.5	19	Nil	22	4	1	4	1	NO
5	18	F	PELNAC®	Nylon 6–0	Nil	Right nasal ala	Compound nevus	1 × 1	29	Nil	22	4	1	4	1	NO
6	75	M	PELNAC®	Nylon 6–0	Nil	Nasal tip	BCC	1 × 1	35	Nil	34	4	1	3	1	NO
7	48	M	PELNAC®	Nylon 6–0	Nil	Right ear concha	BCC	1 × 1	21	Nil	42	5	1	5	2	NO

*BCC* basal cell carcinoma, *HBV* hepatitis B, *HTN* hypertension, *Q1* overall satisfaction about the therapy, *ADM* acellular dermal matrix, *Q2* wound interferes with daily life, *Q3* if you need to receive excision of the other site again, will you choose us, *Scar scale* Vancouver Scar Scale

**Table 4:** Epidemiological data

	Value (Mean ± SD or ratio)
Patient’s age, years	52.28 ± 19.4
Sex (M:F)	3:4
Defect size, cm^2^	0.89 ± 0.28
Healing time, days	23.43 ± 7.41
Satisfaction scale	4.57 ± 0.53
Vancouver Scar Scale	1.2 ± 0.48
Complications	No instances

The defect size ranged from 0.25 to 1 cm^2^ (mean 0.89 ± 0.28 cm^2^). Most of the patients’ wounds healed within 3–4 weeks postoperatively (mean 23.43 ± 7.41 days). The mean satisfaction scale score was 4.57 ± 0.53; no patient underwent secondary procedures for aesthetic modifications. None of the patients were disturbed by their wounds.

All patients were followed up for at least 6 months, and there were no recurrent skin cancers. There were no complications such as infection, hematoma, hyperpigmentation, contracture, or bleeding.

## Discussion

In this report, we describe a simple approach that achieved high patient satisfaction, required less operative time, and led to no recurrences. The locoregional flap was used in these defects in Asians in a previous study and had the same healing time and satisfaction results [10]. Healing took approximately 3–4 weeks in our report, whereas it could take more than 6–8 weeks with the traditional two-stage forehead flap or two-stage reconstruction with an acellular dermal matrix [1, 6, 21]. Reconstruction using a flap takes more time, and close monitoring is necessary [21, 22]. As many Asian patients undergo reconstruction with a flap and experience scars or contractures [10, 11], this approach is meaningful for both the patient and the surgeon.

Compared to two-stage reconstruction with an acellular dermal matrix, single-stage reconstruction can avoid the second operation and skin graft harvest [6]. There was also high patient satisfaction and better aesthetic results with single-stage reconstruction in our report.

The use of an acellular dermal matrix to cover facial defects has been previously proposed. Applebaum et al. reported a case using Integra^®^ and a full-thickness skin graft for a defect of the nasal tip (two-stage); the defect size was 4 cm^2^, the complete surgical process took 1 month, and the wound healed 8 weeks postoperatively [17]. Seth et al. reported on 16 patients with nasal defects who underwent two-stage surgeries with Integra®. The mean size of the wound was 4.0 ± 3.7 cm^2^, the complete surgical process took 1 month, and the wounds healed after 6 weeks [6]. Herein, we had fewer days for healing (mean of 23.43 ± 7.41 days) and smaller wounds, with a mean size of 0.89 ± 0.28 cm^2^.

Burd and Wong reported on 10 Asian patients with facial lesion excisions, of which five were nasal lesions [14]. In these five cases, the average wound size was 2.82 ± 1.61 cm^2^, patients underwent single-stage reconstructions with acellular dermal matrices, and complete healing occurred within 6 weeks [14]. However, these were not cartilage-exposed wounds. We used acellular dermal matrices to achieve similar results in patients with cartilage-exposed wounds.

Other surgeons have encountered complications such as hyperpigmentation and contracture of the wound [6]. In this report, no hyperpigmentation or contracture of the wound was noted, and no patients received further cosmetic surgery for the wound.

**Fig. 3:**
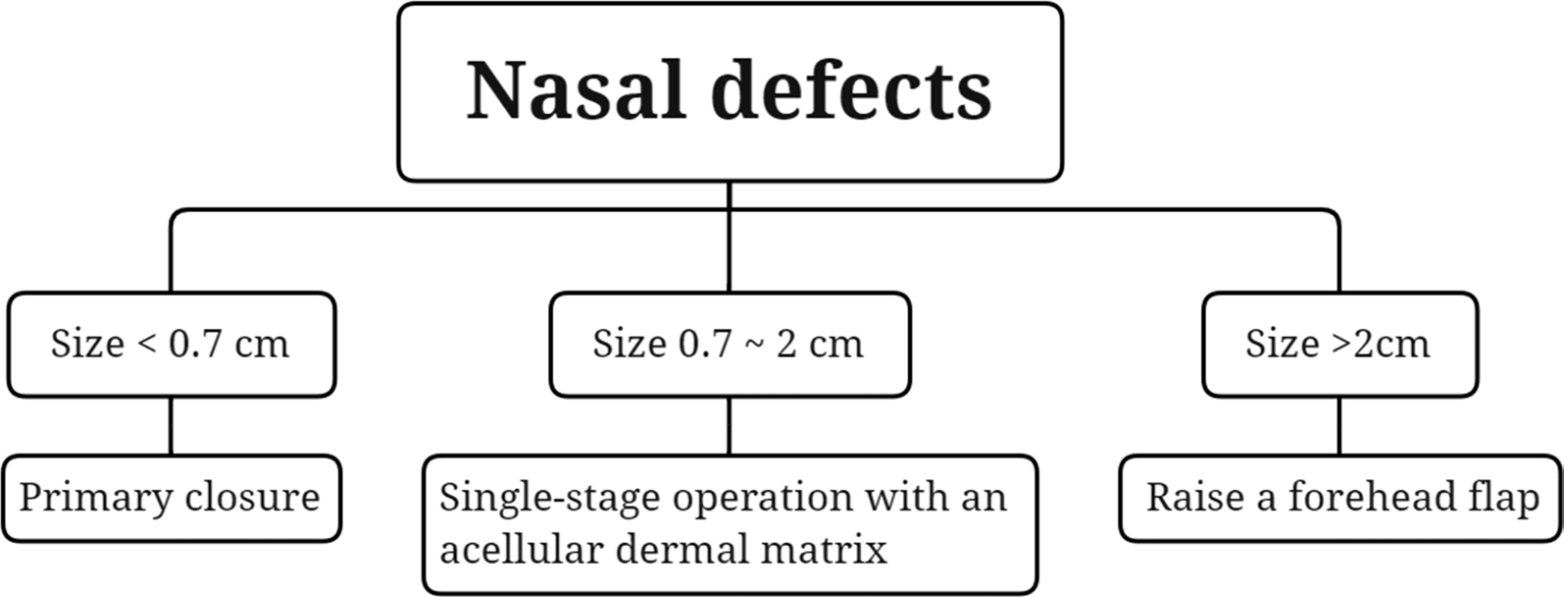
Simple algorithm for the reconstruction of nasal and auricular defects with exposed cartilage in Asians

Koh and Sun attempted to close the defect differently based on the wound size in an Asian cohort; they used primary closure if the wound was < 0.7 cm in size [10]. If the wound size was > 1.2 cm, they used a forehead flap [10, 24]. In this report, similar to the study by Burd and Wong, the surgeon could reconstruct with the acellular dermal matrix if the wound length was 0.5–2.0 cm [10, 14]. Despite the defect having exposed cartilage, a satisfactory result was obtained when the wound size was approximately 1 cm^2^. According to the result of our reports and the previous studies [10, 14, 24], we developed a simple reconstructive algorithm for nasal reconstruction in Asians (Fig. 3).

Other available acellular dermal matrix membranes, such as Integra^®^, have been used for rhinophyma [24]. In the USA, the cost of Integra^®^ varies depending on hospital contracts and usage, and costs approximately 2000 USD for an 8-inch × 10-inch piece [25].

The total cost of reconstruction includes hospitalization, surgical procedures, and outpatient costs. According to Schiavon et al., patients spent more days in the hospital, incurred higher costs of surgical procedures, and had to be followed up more times at outpatient clinics after flap reconstruction; however, the study only considered patients treated for scalp defects with bone exposure. An acellular dermal matrix is less expensive than treatment with free or pedicle flaps [26]. In our report, the patient needed to purchase the acellular dermal matrix at a cost of approximately 14 USD per cm^2^. Patients could receive local anesthesia, be discharged on the same day, and receive follow-up at our outpatient clinic. Benefiting from national health insurance, most surgeons prefer locoregional flaps with general anesthesia and monitor the postoperative condition in the ward for a few days. In Taiwan, general anesthesia costs 125 USD, and the patient is put on observation for a few days (cost of the ward: USD 70 per day). When considering the costs of an operation and the time spent raising the flap, taking the graft, and hospitalization, an acellular dermal matrix is a better and less expensive alternative.

This report has some limitations. First, there were too few cases to analyze the possible complications that may be encountered, and recurrent cancer requires a longer follow-up time. Second, the average size of the wounds was approximately 1 cm^2^; thus, we can only be certain that the use of an acellular dermal matrix is a good choice for defects of this size. Although we had a high acceptance of aesthetic results, different techniques need to be compared simultaneously under the same conditions. Additionally, in our report, we used the Vancouver Scar Scale that is evaluated by the doctors. The Patient and Observer Scar Assessment Scale may be the better choice to evaluate the wound [25]. Additionally, the outcome was evaluated by our team, and evaluation by external experts are needed in future studies. Further comparative studies are required to confirm the efficacy of this approach.

## Conclusion

We used an artificial dermal matrix to cover cartilage-exposed defects without a secondary operation in a series of Asian patients. Based on our results and the results of previous studies, a single-stage operation with an acellular dermal matrix is suitable for wound sizes between 0.7 cm and 2 cm. Additionally, we also did not encounter any instances of hyperpigmentation or contracture. A single-stage operation with an acellular dermal matrix can shorten the treatment duration and offer a more convenient way to treat defects of the nose and ears.

## Data Availability

The data are from and owned by Kaohsiung Municipal United Hospital (KMUH). Based on the agreement between the ethical committee of KMUH and the municipal law of Taiwan for protecting private information, access to the original data is restricted to researchers who are members of KMUH and are accepted by the society. Data are available from the corresponding author upon reasonable request to huangsh63@gmail.com.

## References

[1] Correa BJ, Weathers WM, Wolfswinkel EM, Thornton JF (2013). The forehead flap: the gold standard of nasal soft tissue reconstruction. Semin Plast Surg.

[2] Daya M, Anderson I, Troyer M, Portnof J (2018). Two step reconstruction of traumatic ear skin avulsion using Integra graft. J Stomatol Oral Maxillofac Surg.

[3] Rogers-Vizena CR, Lalonde DH, Menick FJ, Bentz ML (2015). Surgical treatment and reconstruction of nonmelanoma facial skin cancers. Plast Reconstr Surg.

[4] van der Eerden P, Simmons M, Vuyk H (2008). Reconstruction of nasal sidewall defects after excision of nonmelanoma skin cancer: analysis of uncovered subcutaneous hinge flaps allowed to heal by secondary intention. Arch Facial Plast Surg.

[5] Schäfer K, Rudolph C, Cotofana S, Goebeler M, Weyandt G (2018). Large nasal defects with exposed cartilage: the folded transposition flap as an innovative alternative to the paramedian forehead flap. Dermatology.

[6] Seth AK, Ratanshi I, Dayan JH, Disa JJ, Mehrara BJ (2019). Nasal reconstruction using the integra dermal regeneration template. Plast Reconstr Surg.

[7] Cavaliere A, Maisto B, Zaporojan T, Giordano L, Sorbino L, Zaffiro A (2021). Extended rotation flap for reconstruction of partial thickness defects of the tip and nasal ala region: in search of better aesthetic results. JPRAS Open.

[8] Losco L, Bolletta A, Pierazzi DM, Spadoni D, Cuomo R, Marcasciano M (2020). Reconstruction of the nose: management of nasal cutaneous defects according to aesthetic subunit and defect size a review. Medicina (Kaunas).

[9] Schonauer F, Vuppalapati G, Marlino S, Santorelli A, Canta L, Molea G (2010). Versatility of the posterior auricular flap in partial ear reconstruction. Plast Reconstr Surg.

[10] Koh IS, Sun H (2021). A practical approach to nasal reconstruction in Asian patients. Arch Craniofac Surg.

[11] Jin HR, Jeong WJ (2009). Reconstruction of nasal cutaneous defects in Asians. Auris Nasus Larynx.

[12] Cerci FB, Dellatorre G (2016). Paramedian forehead flap combined with hinge flap for nasal tip reconstruction. An Bras Dermatol.

[13] Choi JH, Yoo H, Kim BJ (2021). Nasal alar rim redraping method to prevent alar retraction in rhinoplasty for Asian men: a retrospective case series. Arch Plast Surg.

[14] Burd A, Wong PS (2010). One-stage Integra reconstruction in head and neck defects. J Plast Reconstr Aesthet Surg.

[15] Noda Y, Kuwahara H, Morimoto M, Ogawa R (2018). Reconstruction of anterior neck scar contracture using a perforator-supercharged transposition Flap. Plast Reconstr Surg Glob Open.

[16] Bolletta A, Losco L, Pozzi M, Schettino M, Cigna E (2022). A retrospective study on single-stage reconstruction of the ear following skin cancer excision in elderly patients. J Clin Med.

[17] Applebaum MA, Daggett JD, Carter WL (2015). Nasal tip reconstruction using integra bilayer wound matrix: an alternative to the forehead flap. Eplasty.

[18] Baryza MJ, Baryza GA (1995). The Vancouver Scar Scale: an administration tool and its interrater reliability. J Burn Care Rehabil.

[19] Suzuki S, Matsuda K, Maruguchi T, Nishimura Y, Ikada Y (1995). Further applications of “bilayer artificial skin”. Br J Plast Surg.

[20] Kashimura T, Nagasaki K, Horigome M, Yoshida K, Soejima K (2021). Selection of artificial dermis for shortening treatment period: Integra versus Pelnac. Plast Reconstr Surg Glob Open.

[21] Tiengo C, Amabile A, Azzena B (2012). The contribution of a dermal substitute in the three-layers reconstruction of a nose tip avulsion. J Plast Reconstr Aesthet Surg.

[22] Johnson EL, Danilkovitch A (2018). Nonsurgical management of a large necrotic nasal tip wound using a viable cryopreserved placental membrane. Clin Case Rep.

[23] Calloway HE, Moubayed SP, Most SP (2017). Cost-effectiveness of early division of the forehead flap pedicle. JAMA Facial Plast Surg.

[24] Torresetti M, Scalise A, Di Benedetto G (2019). Acellular dermal matrix for rhinophyma: is it worth it? A new case report and review of literature. Int J Surg Case Rep.

[25] Lee LF, Porch JV, Spenler W, Garner WL (2018). Integra in lower extremity reconstruction after burn injury. Plast Reconstr Surg.

[26] Schiavon M, Francescon M, Drigo D, Salloum G, Baraziol R, Tesei J (2016). The use of integra dermal regeneration template versus flaps for reconstruction of full-thickness scalp defects involving the calvaria: a cost-benefit analysis. Aesthetic Plast Surg.

